# Editorial: Third window syndrome, volume II

**DOI:** 10.3389/fneur.2025.1554737

**Published:** 2025-03-20

**Authors:** P. Ashley Wackym, Carey D. Balaban, Konstantina M. Stankovic, Tetsuo Ikezono, Todd M. Mowery

**Affiliations:** ^1^Department of Head and Neck Surgery & Communication Sciences, Rutgers Robert Wood Johnson Medical School, New Brunswick, NJ, United States; ^2^Department of Otolaryngology, University of Pittsburgh, Pittsburgh, PA, United States; ^3^Department of Otolaryngology – Head and Neck Surgery, Stanford University School of Medicine, Palo Alto, CA, United States; ^4^Department of Otolaryngology and Neuro-Otology, Saitama Medical University Hospital, Saitama, Japan

**Keywords:** dizziness, headache, migraine, otic capsule dehiscence, perilymph fistula, superior semicircular canal dehiscence, third window syndrome, vestibular migraine

This Research Topic, *Third window syndrome volume II*, compiles the latest discoveries on the mechanisms underlying the spectrum of symptoms and dysfunction associated with Third Window Syndrome. It presents novel diagnostic tools and interventions aimed at identifying and resolving this condition.

Nearly a century ago, Tullio described the physiologic outcomes of creating a third mobile window in the semicircular canals of pigeons (1, 2). Although third mobile windows have been identified subsequently at other sites in the labyrinth, the “Tullio phenomenon” eponym Is applied generally to sound-induced dizziness and/or nystagmus. Superior semicircular canal dehiscence is clearly the best-known and most thoroughly characterized type of a third mobile window. In 1998, Minor and coworkers first reported sound- and/or pressure-induced vertigo due to bone dehiscence of the superior semicircular canal, confirmed on CT scans (3). Minor later distinguished both an inner ear conductive hearing loss (i.e., bone-conduction hyperacusis), and a reduced cervical vestibular myogenic potential (cVEMP) threshold with increased amplitude responses in patients with superior semicircular canal dehiscence. While the clinical phenotype associated with the superior semicircular canal dehiscence is well-recognized; third window syndrome with the same clinical phenotype has been reported in patients without radiographic evidence of a frank superior semicircular canal dehiscence (4–9). Such a CT-negative third window syndrome is associated with an inner ear conductive hearing loss and an abnormally reduced cVEMP threshold, among other objective findings typically found in patients with superior semicircular canal dehiscence (4–9). The more general term of Third Window Syndrome has gained acceptance recently because the same spectrum of symptoms, signs on physical examination and audiological diagnostic findings are associated with mobile third windows at different otic capsule loci. These locations includes superior semicircular canal dehiscence, superior semicircular canal dehiscence-superior petrosal vein dehiscence, superior semicircular canal dehiscence-sub-arcuate artery dehiscence, lateral semicircular canal-superior semicircular canal ampulla dehiscence, posterior semicircular canal dehiscence, posterior semicircular canal-endolymphatic sac dehiscence, posterior semicircular canal-jugular bulb dehiscence, cochlea-internal carotid artery dehiscence, cochlea-internal auditory canal dehiscence, cochlea-facial nerve dehiscence, modiolus, perilymph fistula, vestibule-middle ear dehiscence, lateral semicircular canal-facial nerve dehiscence, wide vestibular aqueduct in children, posttraumatic hypermobile stapes footplate and in patients with CT negative Third Window Syndrome [review see (4–9)]. Pathological third mobile window at an otic capsule defect is the common structural finding in all of these conditions.

Over the past 70 years, our understanding of Third Window Syndrome has advanced significantly. Contributions from objective diagnostic studies, descriptions of clinical features, assessment of clinical outcomes with validated survey instruments, and neuropsychology testing have expanded the disciplinary scope diagnosis and treatment of these disorders. One restricted to the hallmark symptoms of sound-induced otolithic dysfunction (dizziness) and autophony, consequences of Third Window Syndrome are recognized to encompass manifestations of anxiety, migraine, spatial disorientation and cognitive dysfunction. The collection of papers featured in this Research Topic offers valuable insights for both scientists and clinicians working in the fascinating fields of peripheral vestibular dysfunction and its related central pathophysiology.

This Research Topic was truly global in effort and representation with four continents: Asia, Australia/Oceania, Europe, and North America. However, three were not represented: Africa, Antarctica, and South America. There were five countries represented: USA; Japan, France, Canada, and New Zealand. There were 68 authors.

In this editorial, we present an overview of the 12 published studies included in this Research Topic, organized into the following categories: New Animal Model; Diagnostic Studies and New Diagnostic Tools; Sites of Dehiscence; Surgical Advances; and Surgical Outcomes.

## New animal model

Wackym et al. have developed and reported a gerbil model of superior semicircular canal dehiscence, displaying reversible diagnostic findings that are characteristic of patients with the disorder, such as an inner ear conductive hearing loss and increased amplitude cervical positive vestibular evoked myogenic potentials. Using this animal model, Mowery et al. demonstrated reversible impairments in specific auditory and visual behavioral tasks that assess decision-making performance. Specific and reversible cognitive deficits were associated with vestibular dysfunction in the presence of the otic capsule deficit. Specifically, the animals with a surgically-induced, superior semicircular canal dehiscence displayed reversible deficits in a spatial two-alternative forced-choice task, in which they must choose between a left or right option to receive a food reward. Together, these findings show how an otic capsule defect can disrupt normal decision-making behaviors. Most recently, Hong et al. used the same gerbil superior semicircular canal dehiscence model to confirm that aberrant asymmetric vestibular output results in reversible balance impairments, similar to those observed in patients after superior semicircular canal dehiscence plugging surgery. Together, these findings show how a unilateral mobile third window can disrupt normal cognitive functions and behaviors, and the methodology used to establish the gerbil animal model can be employed in other species to systematically investigate the influence of vestibular function on peripheral ear and central cognitive processing.

## Diagnostic studies and new diagnostic tools

Third Window Syndrome has emerged as a significant clinical diagnosis, benefiting thousands of patients globally through its discovery and the development of effective treatments. Moreover, this syndrome serves as valuable pathologic phenomenon that enhances our understanding of the physiology of the vestibular end organs, and aids in the development and refinement of diagnostic tools that probe various aspects of the vestibular system.

Kileny et al. studied the diagnostic utility of electrocochleography (EcochG) relative to the inner ear conductive hearing loss in 20 patients with confirmed superior semicircular canal dehiscence. Eleven patients had unilateral superior semicircular canal dehiscence, and nine patients had bilateral superior semicircular canal dehiscence demonstrated by high-resolution temporal bone CT scanning. There were 29 ears with superior semicircular canal dehiscence and 11 normal ears included in their study. They found that all confirmed superior semicircular canal dehiscence ears presented with an abnormal EcochG summating potential to action potential (SP/AP) ratio value and that there was a statistically significant difference between normal and dehiscent ears. There was no statistically significant relationship between inner ear conductive hearing loss air-bone gap and SP/AP ratio in the ears diagnosed with superior semicircular canal dehiscence. Further, there was no significant difference in the inner ear conductive hearing loss air-bone gap at three frequencies between the normal and dehiscent ears. The authors concluded that EcochG remains a valuable diagnostic tool for superior semicircular canal dehiscence. They also emphasized that the variability in the air-bone gap associated with inner ear conductive hearing loss should not drive the decision to include EcochG in the diagnostic test battery for patients suspected of having this condition.

Ito et al. reported the case of a 27-year-old female who complained of hearing disturbance in her right ear and recurrent vertigo after sudden onset of hearing loss with vertigo. She had reduced vestibular function in the affected ear demonstrated by caloric testing and video head impulse testing. The innovative diagnostic testing reported used a contrast-enhanced MRI technique using hybrid of reversed positive image of endolymph signal and a negative image of perilymph signal which they interpreted as a collapsed endolymphatic space. After failed medical management and with persistent vestibular symptoms, an exploratory right tympanotomy was performed and both the round and oval windows were sealed with connective tissue. The patient's vestibular symptoms improved rapidly after surgery, accompanied by imaging improvement of the collapsed endolymphatic space in a postoperative contrast-enhanced MRI. A unilateral weakness persisted in the caloric test postoperatively, but the VOR gain on the vHIT improved to normal on the right side. Thus, these findings are consistent with concept a collapsed endolymphatic space may contribute to recurrent symptoms caused by a perilymph fistula. They speculated that a ruptured Reissner membrane contributed to the collapsed endolymphatic space and further hypothesized that sealing the fistula resulted in normalization of the perilymph pressure by promoting healing of the ruptured Reissner membrane. This case added to the existing literature on the occurrence of the “*double-membrane break syndrome.”*

Kubota et al. investigated the diagnostic performance of endoscopic middle ear examination compared to testing for cochlin-tomoprotein test (newly developed perilymph specific protein detection test) in diagnosing idiopathic perilymphatic fistula. Diagnosing this condition is particularly difficult when patients present with sudden sensorineural hearing loss or vestibular symptoms without any clear prior incidents. The study examined five patients who initially received intratympanic dexamethasone treatment for sudden sensorineural hearing loss, during which a cochlin-tomoprotein test was also conducted. For those who did not respond to corticosteroids, endoscopic perilymph fistula repair was performed, sealing the oval and round windows using connective tissue and fibrin glue. The researchers assessed cochlin-tomoprotein levels preoperatively and intraoperatively, findings from endoscopic surgery, and changes in hearing and vestibular symptoms both before and after the procedure. Results showed varied cochlin-tomoprotein levels: three patients had positive pre-operative and intermediate intraoperative values, one patient had positive preoperative but negative intraoperative values, and one showed negative preoperative but positive intraoperative values. No patient displayed clear endoscopic evidence of a fistula or perilymph leakage during surgery. Hearing improvement was minimal, with only two patients showing slight recovery. Of the four patients who experienced disequilibrium before surgery, two reported resolution of these symptoms post-operatively. The study concluded that a positive cochlin-tomoprotein test can provide confirmation of perilymph fistula in cases lacking obvious intraoperative findings.

## Sites of dehiscence

Ionescu et al. published an interesting series of complex superior semicircular canal dehiscence patients whose outcomes were not as successful as expected. They initially considered both errors in surgical repair technique and the possibility of co-existing sites of otic capsule defects as factors contributing to the poorer-than-expected clinical outcomes. A review of the radiological and clinical files, they discussed possible surgical technique issues in one case and the likelihood of other undetected dehiscence sites. They completed a retrospective analysis of high-resolution temporal bone CT scans from 157 patients (314 ears), collected over a 5-year period, to examine the prevalence of both symptomatic and asymptomatic Third Window Syndrome. They detected multiple suspected sites of otic capsule dehiscence in the ipsilateral ear in 29 of 157 patients (18.47%).

These findings were similar to those reported in 2019 by Wackym et al. in the original Research Topic focused on Third Window Syndrome. A dehiscence was found in 463 temporal bones of ears with Third Window Syndrome symptoms (57.7% [463/802]). The single sites included superior semicircular canal dehiscence, near-superior semicircular canal dehiscence, CT negative Third Window Syndrome, cochlea-facial nerve, cochlea-internal auditory canal, wide vestibular aqueduct, lateral semicircular canal, modiolus, and posterior semicircular canal, superior semicircular canal dehiscence and superior petrosal sinus, superior semicircular canal dehiscence, and subarcuate artery dehiscence. After excluding temporal bones from cases with no CT evidence of a dehiscence, the remaining 402 bones included 366 cases (91.0%) with a single temporal bone dehiscence site. Multiple sites of dehiscence were observed less frequently, For example, a two site dehiscence was observed in 9.38% of cases (superior semicircular canal dehiscence and cochlea-facial nerve dehiscence, cochlea-facial nerve dehiscence and cochlea-internal auditory canal, cochlea-facial nerve dehiscence and wide vestibular aqueduct canal dehiscence, superior semicircular and cochlea-internal auditory canal, superior semicircular canal dehiscence and posterior semicircular canal-jugular bulb). The most prevalent combination. A superior semicircular canal dehiscence and cochlea-facial nerve dehiscence, accounted for 6% of all cases. The combination of (30/502). Thus, the Ionescu et al. study and the earlier Wackym et al. study agree that the prevalence of multiple-site findings is important to consider when faced with recurrent or incompletely resolved Third Window Syndrome symptoms after plugging a superior semicircular canal dehiscence. Both studies also emphasize the prudence of careful assessment of the potential additional dehiscence sites prior to determining the surgical approach for managing superior semicircular canal dehiscence.

In this Research Topic, Seo et al. reported a case of a microfissure near the round window niche that communicated between middle ear and the ampulla of the posterior semicircular canal. They reported the first case of a patient successfully treated with perilymph fistula repair surgery, presenting with ipsilateral hearing loss, tinnitus described as a flowing-water sound, and a floating sensation triggered by pressing the left tragus, which was caused by an inner ear microfissure. An exploratory tympanotomy was performed 8 days after onset of his symptoms, revealing intraoperative findings of a microfissure and an accumulation of clear fluid in the floor of the round window niche. The site of leakage was sealed with connective tissue. One month after surgery, clinical improvement in his hearing and disequilibrium suggested that the microfissure contributed to his auditory and vestibular symptoms.

## Surgical advances

Sawada et al. described “a multilayer round window reinforcement technique” in managing patients with superior semicircular canal dehiscence (SSCD). This technique involved making an incision within the external auditory canal, and collecting loose areolar tissue, which was compressed using a fascia press and cut into 3 to 5-mm pieces. Cartilage and perichondrium were obtained from the tragus, then thinned and shaped into approximately 2–3 mm circular sections. A CO_2_ laser was employed to remove mucosa from the area around the round window niche. A thin piece of cartilage with perichondrium was positioned within the bony overhang, with the perichondrium side facing the round window to prevent damage to its membrane. Small cartilage fragments, about 0.25 mm in size, were added around the initial cartilage to fill gaps and stabilize the structure. Thinned connective tissue was layered over this structure, adhering to the exposed bone, while additional cartilage pieces were placed on top to secure the reinforcement. Finally, fibrin glue was used to hold everything in place.

To illustrate the outcomes of this technique, Sawada et al. shared two case studies where their “multilayer round window reinforcement technique” was applied to patients with SSCD. The procedure led to significant symptom relief, including diminished autophony, reduced hypersensitivity to bone-conducted sounds, decreased pulsatile tinnitus, and fewer vestibular disturbances triggered by sound or pressure. Patients also reported a lessening of aural fullness. Post-surgical assessments indicated notable improvements in scores from the Dizziness Handicap Inventory, Vertigo Symptom Scale Short Form, and the Niigata Persistent Postural-Perceptual Dizziness Questionnaire.

Altamami et al. developed a manual neuronavigation technique to more consistently identify the superior semicircular canal and the site of dehiscence in superior semicircular canal dehiscence. While a computer-assisted neuronavigation system is useful to precisely identify the location of a superior semicircular canal dehiscence, there are several limitations regarding the cost of purchasing these systems and the need to charge patients for their use during the surgery. Additional limitations of computer-assisted neuronavigation systems are that more surgical time is required to set up, calibrate and use during middle cranial fossa superior semicircular canal dehiscence plugging, which is exacerbated when the surgeon's experience is limited. The study reported by Altamami et al. demonstrated a simple manual neuronavigation technique that can help neurotologic surgeons identify the superior semicircular canal dehiscence accurately and efficiently. They demonstrated that the use of “*line A”* on the preoperative high-resolution temporal bone CT axial cut, which measures the distance from the superior semicircular canal dehiscence to the lateral cortical part of the supra-auricular squamous bone, provides a precise distance that can be measured during surgery. These findings were supplemented by two instructional videos that explained and demonstrated the technique.

## Surgical outcomes

Matsuda et al. presented a retrospective analysis involving 22 patients who underwent surgical treatment for perilymph fistula after conservative treatment had failed. The study explored case characteristics and assessed the effectiveness of the procedure in alleviating both vestibular and auditory symptoms. It was observed that patients with prior triggering events had a significantly shorter duration between symptom onset and surgery. Post-operatively, 82% of the patients experienced substantial relief from vestibular symptoms within a week, even among those with long-standing conditions. Although the study lacked a control group, the marked improvement in vestibular function and the significant reduction in Dizziness Handicap Inventory scores suggest that these outcomes were likely a result of the surgical intervention. Additionally, early surgical intervention showed improvements in hearing, with some positive effects also noted in cases with delayed surgery. By utilizing cochlin-tomoprotein, a perilymph-specific protein, as a biomarker, the study confirmed that the presence of a perilymph fistula contributed to both balance and auditory issues in these patients. Furthermore, the authors introduced a new hypothesis, referred to as the “Hyperactive Utricular Movement Theory,” suggesting that chronic imbalance in these cases may result from increased utricular mobility rather than endolymphatic hydrops.

Benchetrit et al. sought to identify predictors of symptom persistence after surgical management of superior semicircular canal dehiscence. They conducted a retrospective study of 132 ears in 126 patients who underwent superior semicircular canal dehiscence plugging via the middle cranial fossa or transmastoid approach. The authors used a previously published standardized symptomatology questionnaire from their preoperative and postoperative visits. The questionnaire asked patients to identify if their most bothersome complaint is hearing-related or balance-related. Binary (yes/no) responses were recorded for the subjective experience of 11 auditory symptoms [hearing loss, aural fullness, pulsatile tinnitus, non-pulsatile tinnitus, autophony (hearing your voice too loudly), hyperacusis, hearing your voice echo, hearing your footsteps, hearing your eyeballs moving or hearing hair brushing, or shaving sounds too loudly] and eight vestibular symptoms (general dizziness, sense of imbalance, Tullio phenomenon, straining causing dizziness, physical activity causing dizziness, blowing your nose/sneezing/coughing causing dizziness, oscillopsia, and positional dizziness). Information regarding postoperative resolution of primary (most bothersome) symptom complaint was obtained from reviewing the electronic medical record and stratified to the categories of resolved, improved and persisted. The preoperative vs. post-operative survey results, demographic and clinical characteristics, operative characteristics, audiometric data and cervical vestibular evoked myogenic potential (cVEMP) thresholds were compared via univariate χ^2^ and multivariate binary logistic regression analyses between those patients reporting full post-operative resolution of symptoms and persistence of one or more symptoms. The authors found that of the 132 ears in 126 patients, 119 patients (90.2%) reported postoperative resolution (*n* = 82, 62.1%) or improvement (*n* = 37, 28.0%) of primary (most bothersome) symptoms, while 13 patients (9.8%) reported persistence of primary symptoms. The median (interquartile range) and range between surgery and questionnaire completion were 9 months (4–28), 1–124 months, respectively. Analyzing all symptoms (primary and non-primary) 69 (52.3%) and 68 (51.1%) patients reported complete postoperative auditory and vestibular symptom resolution, respectively. They found that the most likely persistent symptoms included imbalance (33/65, 50.8%), positional dizziness (7/20, 35.0%), and oscillopsia (44/15, 26.7%). Factors associated with persistent auditory symptoms included history of seizures (0% vs. 7.6%, *p* = 0.023), auditory chief complaint (50.0% vs. 70.5%), higher PTA (mean 19.6 vs. 25.1 dB, *p* = 0.043) and higher cervical vestibular evoked myogenic potential (cVEMP) thresholds at 1,000 Hz (mean 66.5 dB vs. 71.4 dB, *p* = 0.033). A migraine diagnosis (14.0% vs. 41.9%, *p* < 0.010), bilateral radiologic superior semicircular canal dehiscence (17.5% vs. 38.1%, *p* = 0.034), and revision cases (0.0% vs. 14.0%, *p* = 0.002) were associated with persistent vestibular symptoms. They also found that neither superior semicircular canal dehiscence size nor location were significantly associated with symptom persistence (*p* > 0.05). The authors concluded that surgical plugging of a superior semicircular canal dehiscence results in a meaningful reduction in the majority of auditory and vestibular symptoms; however, the persistence of certain, mostly non-primary, symptoms, and the identification of potential associated factors including migraines, pure tone average thresholds, cVEMP thresholds, bilateral superior semicircular canal dehiscence, and patients representing revision cases underscore the need for individualized patient counseling and management strategies.

## Conclusions

In this Editorial, we highlight the 12 published studies included in this Research Topic and organized them in the following categories: New Animal Model; Diagnostic Studies and New Diagnostic Tools; Sites of Dehiscence; Surgical Advances; and Surgical Outcomes.

Research on new diagnostic tools indicates that Third Window Syndrome can provide valuable insights into the mechanisms of the inner ear. There are three key symptoms and physical signs that are essential for identifying Third Window Syndrome, regardless of specific location of the dehiscence: (1) sound-induced dizziness; (2) hearing internal sounds; and (3) hearing or feeling low frequency tuning forks in an involved ear when applied to a patient's knee or elbow. The sound-induced auditory and vestibular activity is distinct from other balance disorders because the transient vestibular afferent activity is uncoupled from motion of the head or body in space (allocentric reference frame) or from motion of the environment around the head and body (egocentric reference frame). The sound-induced auditory and vestibular activity will also be uncorrelated with contextual visual, somesthetic and interoceptive sensory information and on-going (or planned) motor activity. While the studies examining cognitive and spatial orientation in Third Window Syndrome shed light on important cognitive outcome measures for researching patients with vestibular impairments, neither their measures nor validated survey instruments for symptoms—such as the Dizziness Handicap Inventory—are specifically tailored to account for the distinctive perceptual incongruities present in Third Window Syndrome compared to other conditions. Although current tools may be useful for monitoring patient outcomes in the management of Third Window Syndrome, there is potential for improvement and refinement. The studies included in this Research Topic provided useful conceptual and state-of-the-art frameworks to better understand peripheral bases for the signs and symptoms of common forms of Third Window Syndrome. In addition, a series of basic research studies developing a new animal model of superior semicircular canal dehiscence creates the opportunity to study the fundamental neuroanatomic circuitry underlying the changes in cognitive dysfunction and other central nervous system phenomena that patients with Third Window Syndrome experience. These frameworks are essential for designing specific diagnostic tests and new, potentially therapeutic approaches. Finally, rare and newly identified sites of dehiscence creating a third mobile window were presented and surgical advances to manage various sites resulting in Third Window Syndrome were reported. Together, these 12 studies provide a comprehensive overview of our current knowledge, as well as gaps that remain in understanding, diagnosing and managing of patients with Third Window Syndrome.
